# Supplementary figures and images for: Integrating multi-omics data reveals function and therapeutic potential of deubiquitinating enzymes
Source: eLife. 2022 Jun 23;11:e72879. doi: 10.7554/eLife.72879 (PMC9225015; doi:10.7554/eLife.72879)

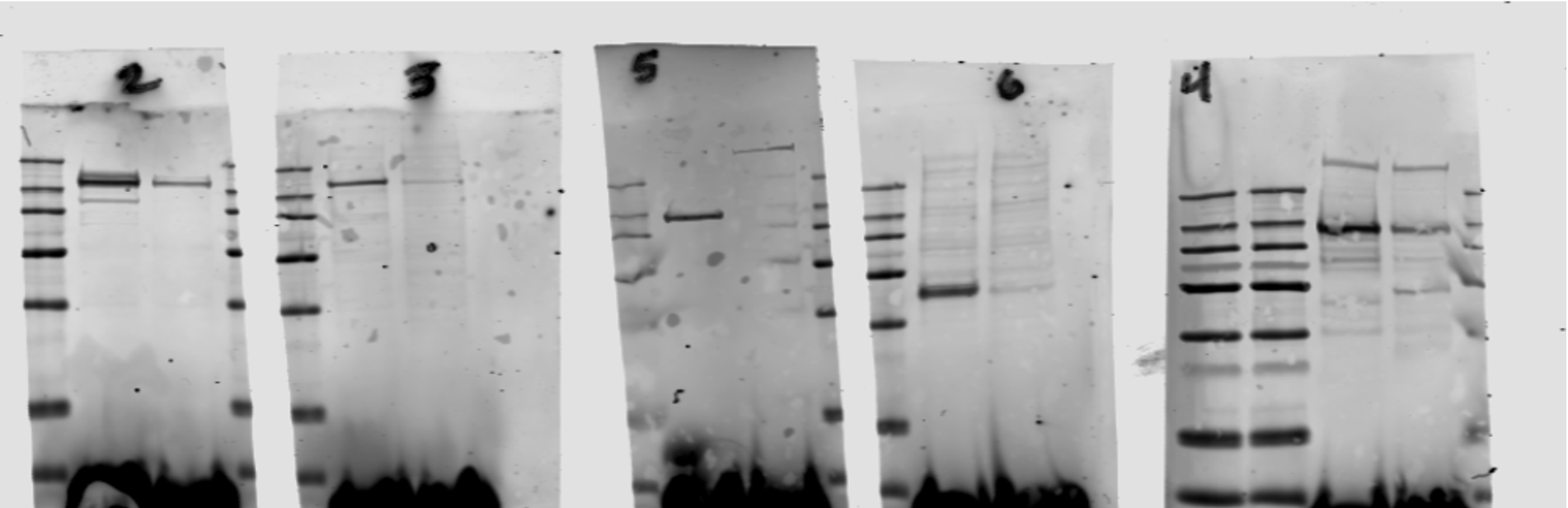

Supplement: Figure 2—figure supplement 1—source data 1. [file elife-72879-fig2-figsupp1-data1.png]

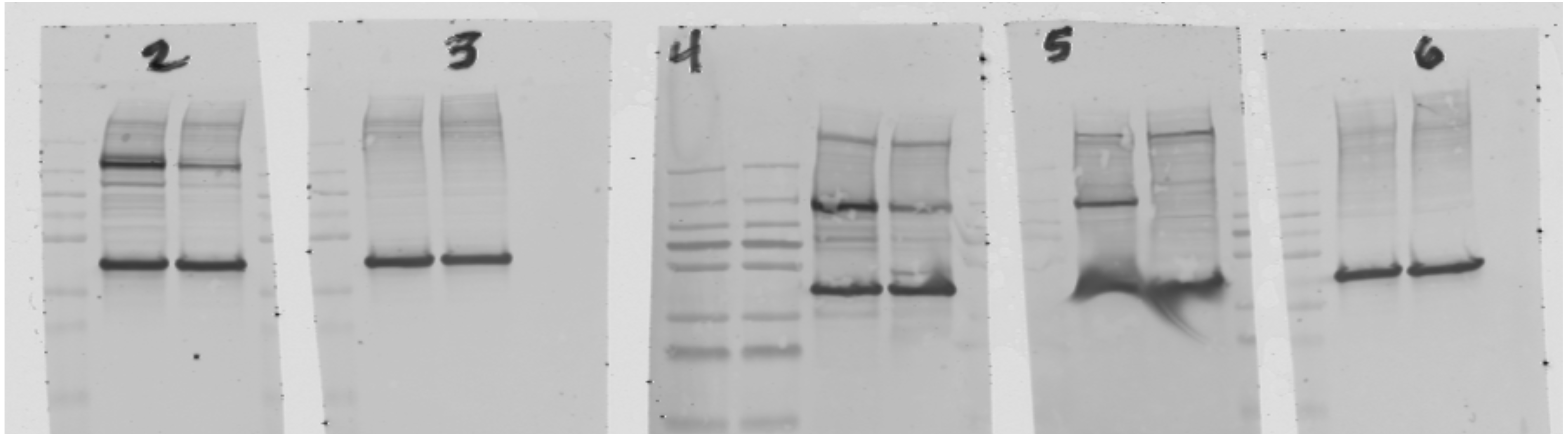

Supplement: Figure 2—figure supplement 1—source data 2. [file elife-72879-fig2-figsupp1-data2.png]

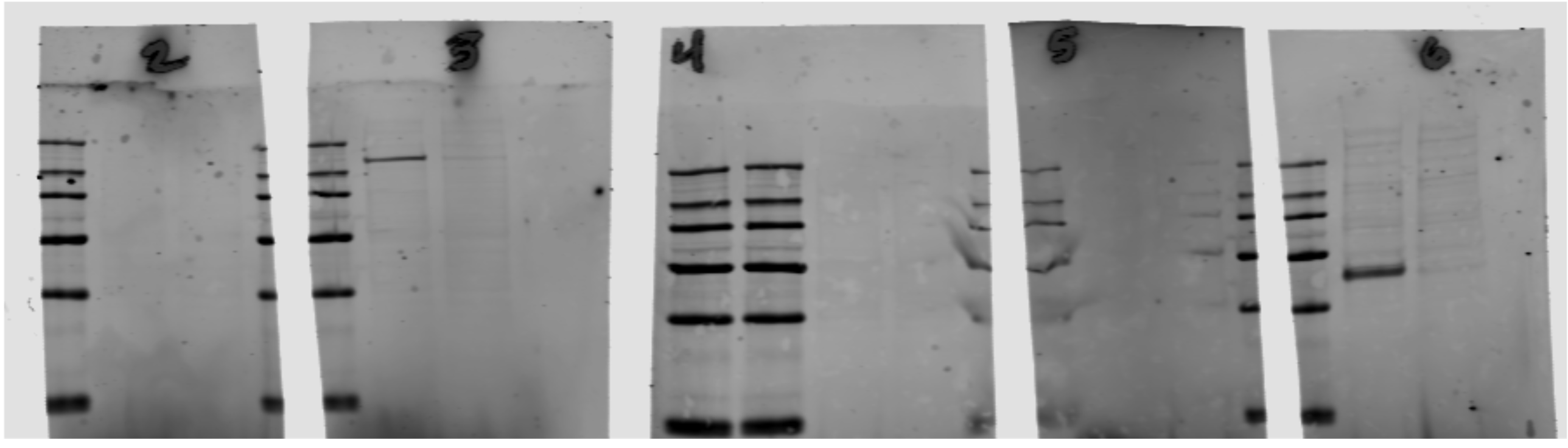

Supplement: Figure 2—figure supplement 1—source data 3. [file elife-72879-fig2-figsupp1-data3.png]

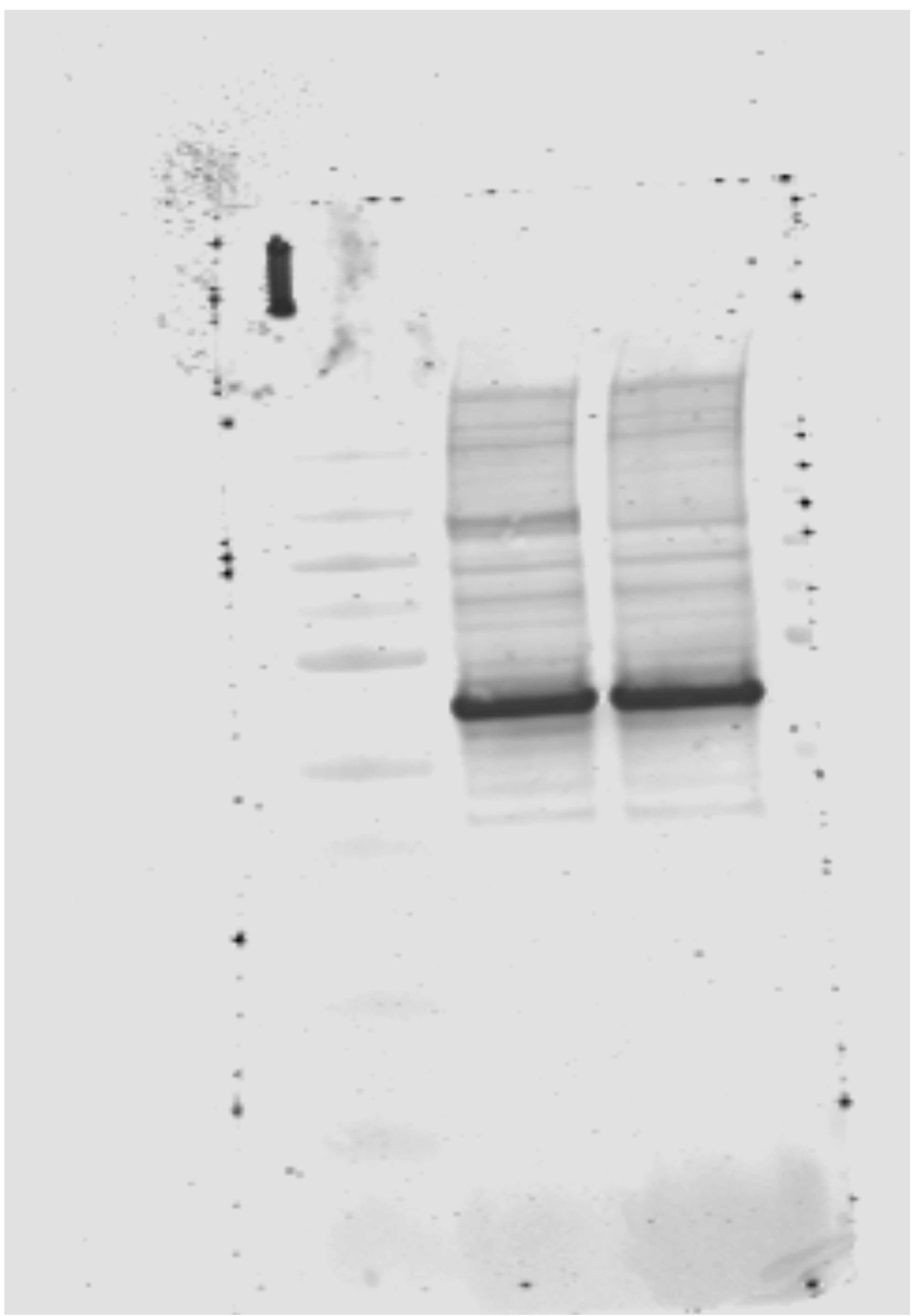

Supplement: Figure 2—figure supplement 1—source data 4. [file elife-72879-fig2-figsupp1-data4.png]

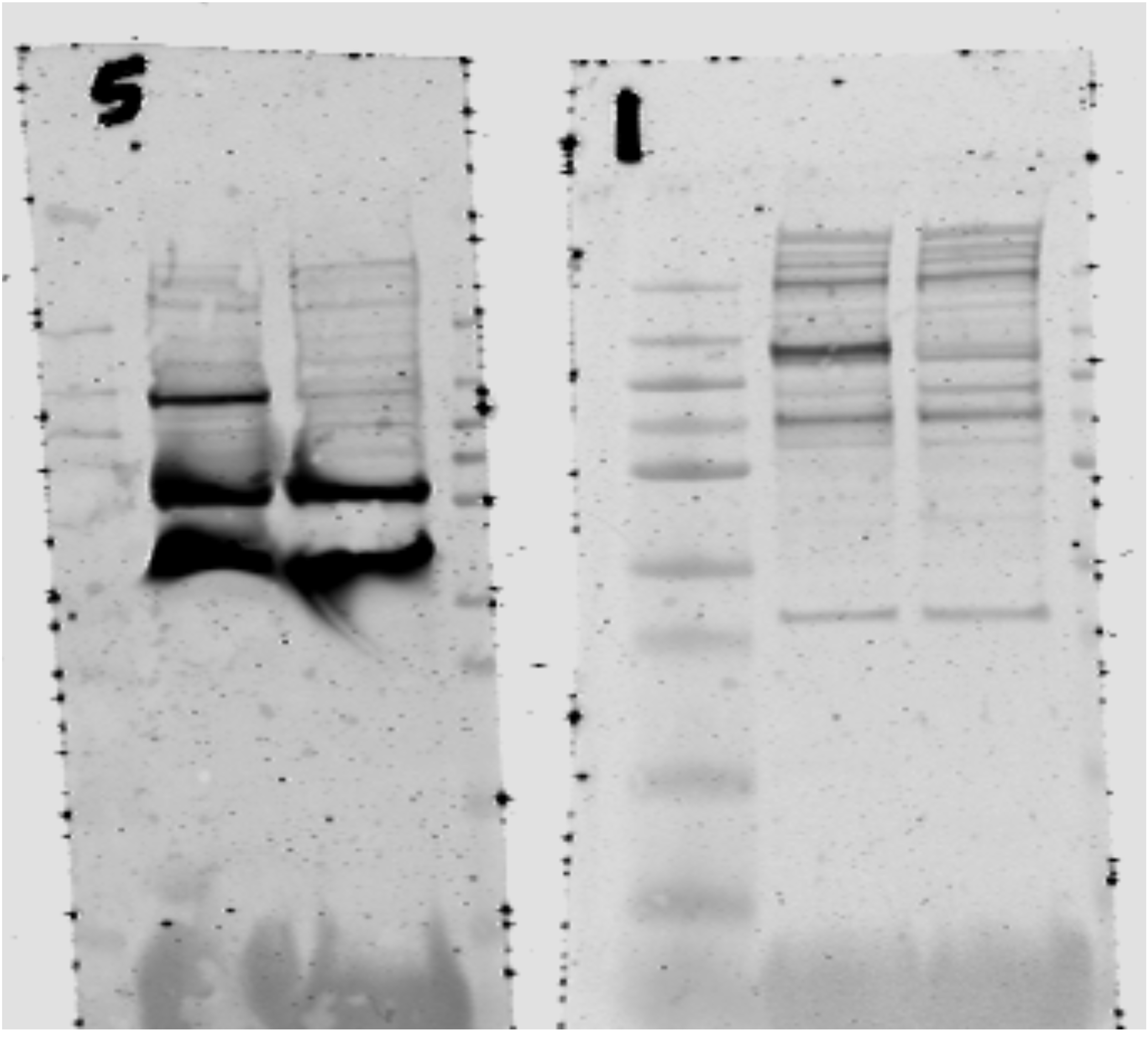

Supplement: Figure 2—figure supplement 1—source data 5. [file elife-72879-fig2-figsupp1-data5.png]

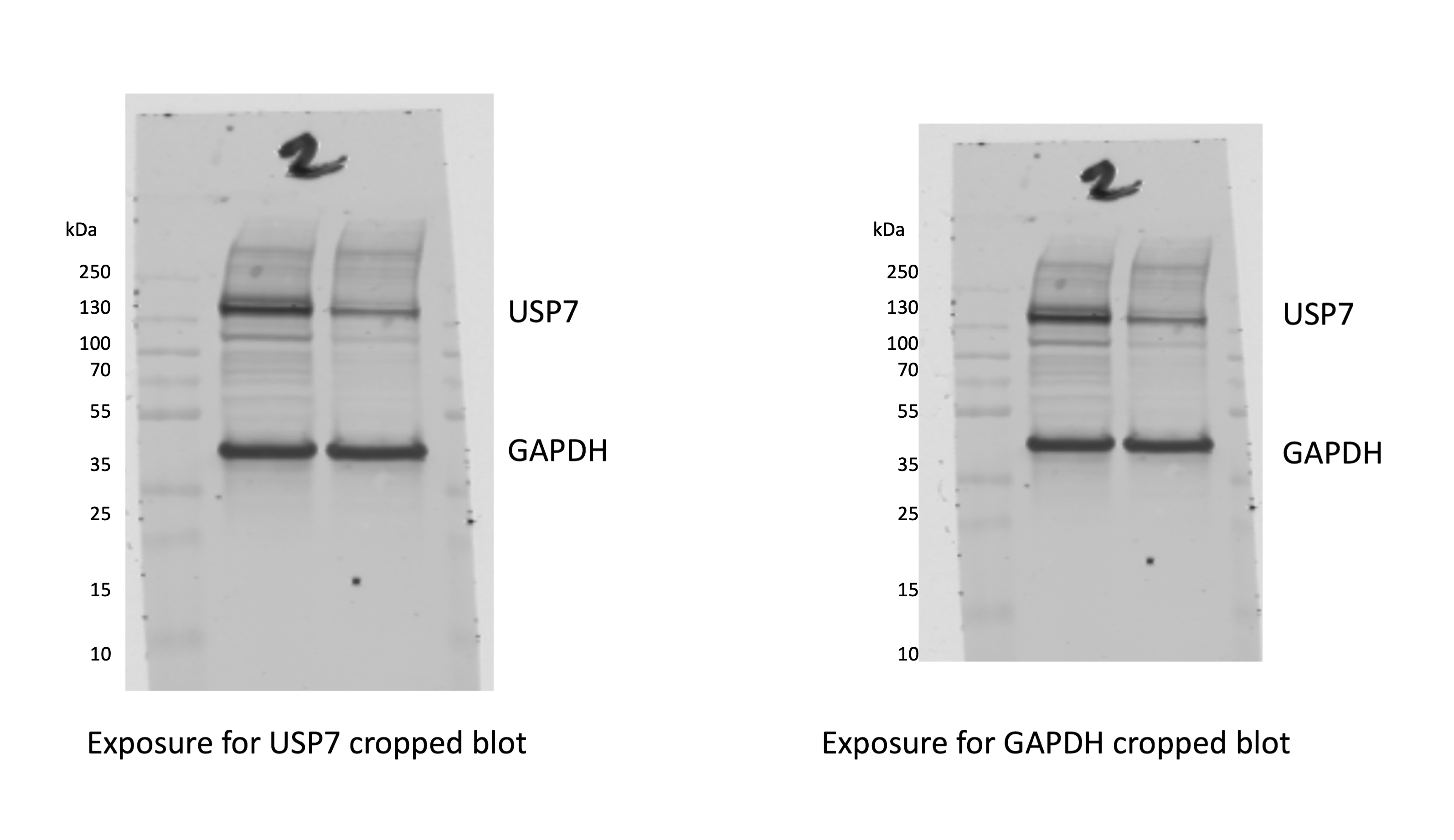

Supplement: Figure 2—figure supplement 1—source data 6. [file elife-72879-fig2-figsupp1-data6.png]

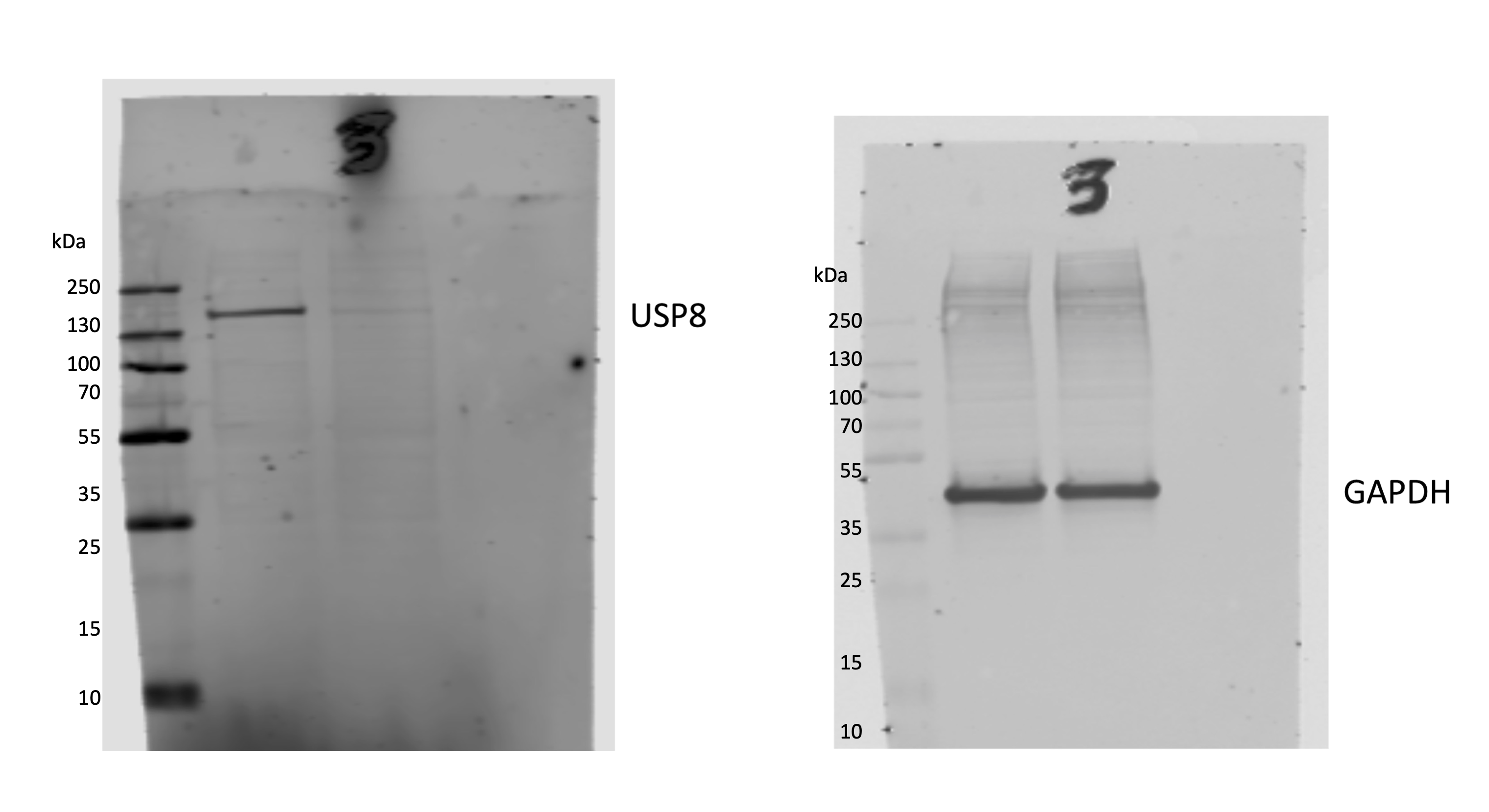

Supplement: Figure 2—figure supplement 1—source data 7. [file elife-72879-fig2-figsupp1-data7.png]

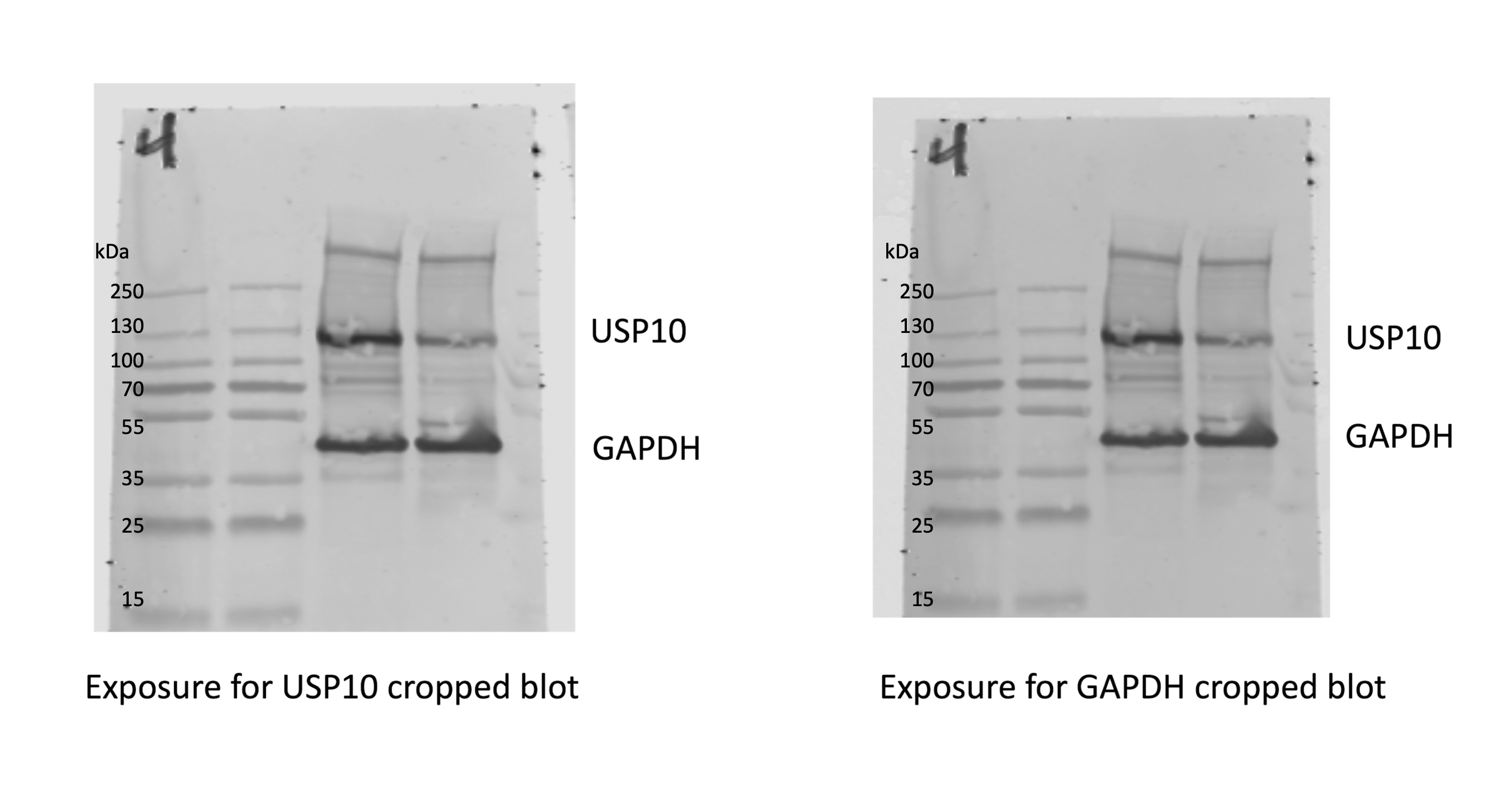

Supplement: Figure 2—figure supplement 1—source data 8. [file elife-72879-fig2-figsupp1-data8.png]

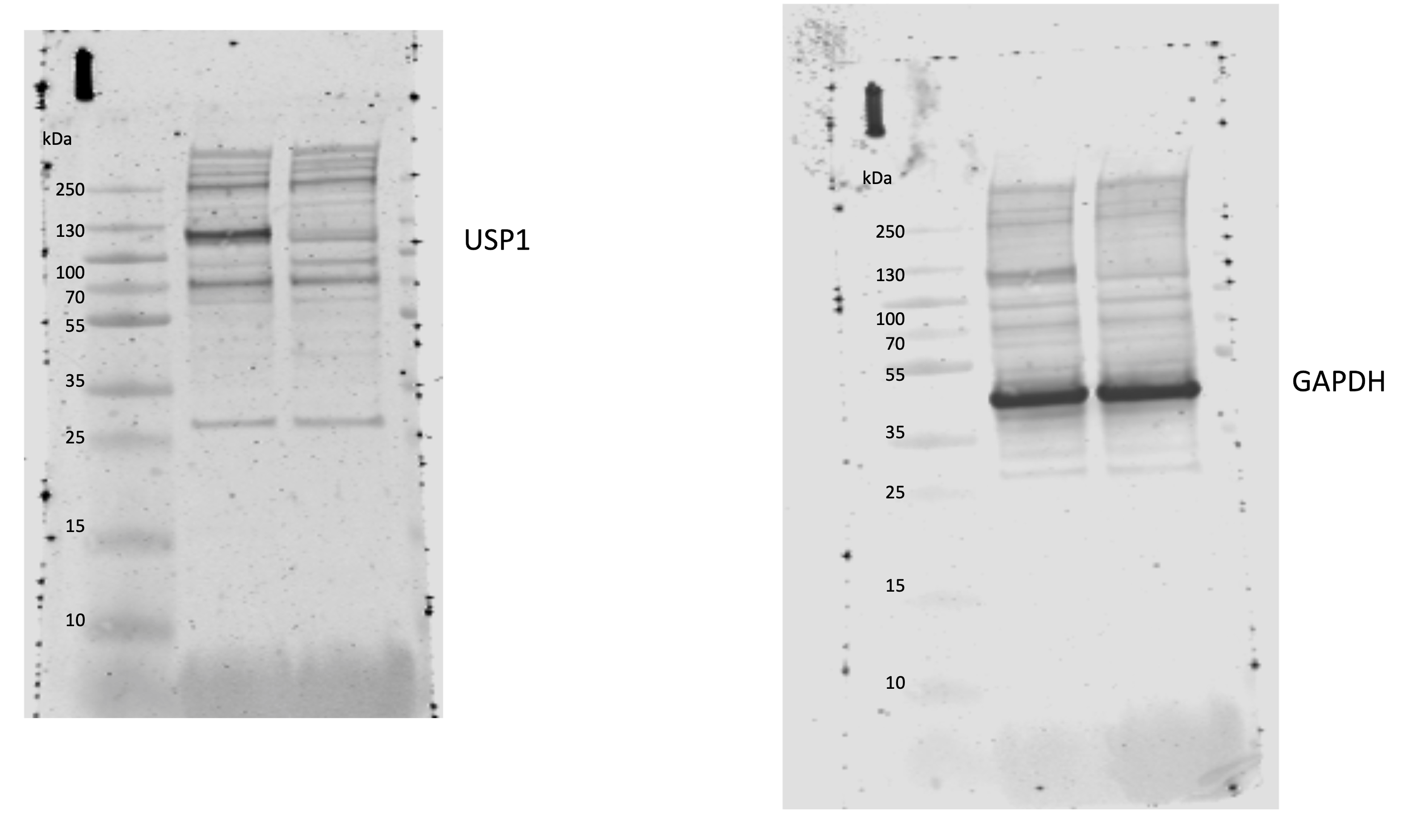

Supplement: Figure 2—figure supplement 1—source data 9. [file elife-72879-fig2-figsupp1-data9.png]

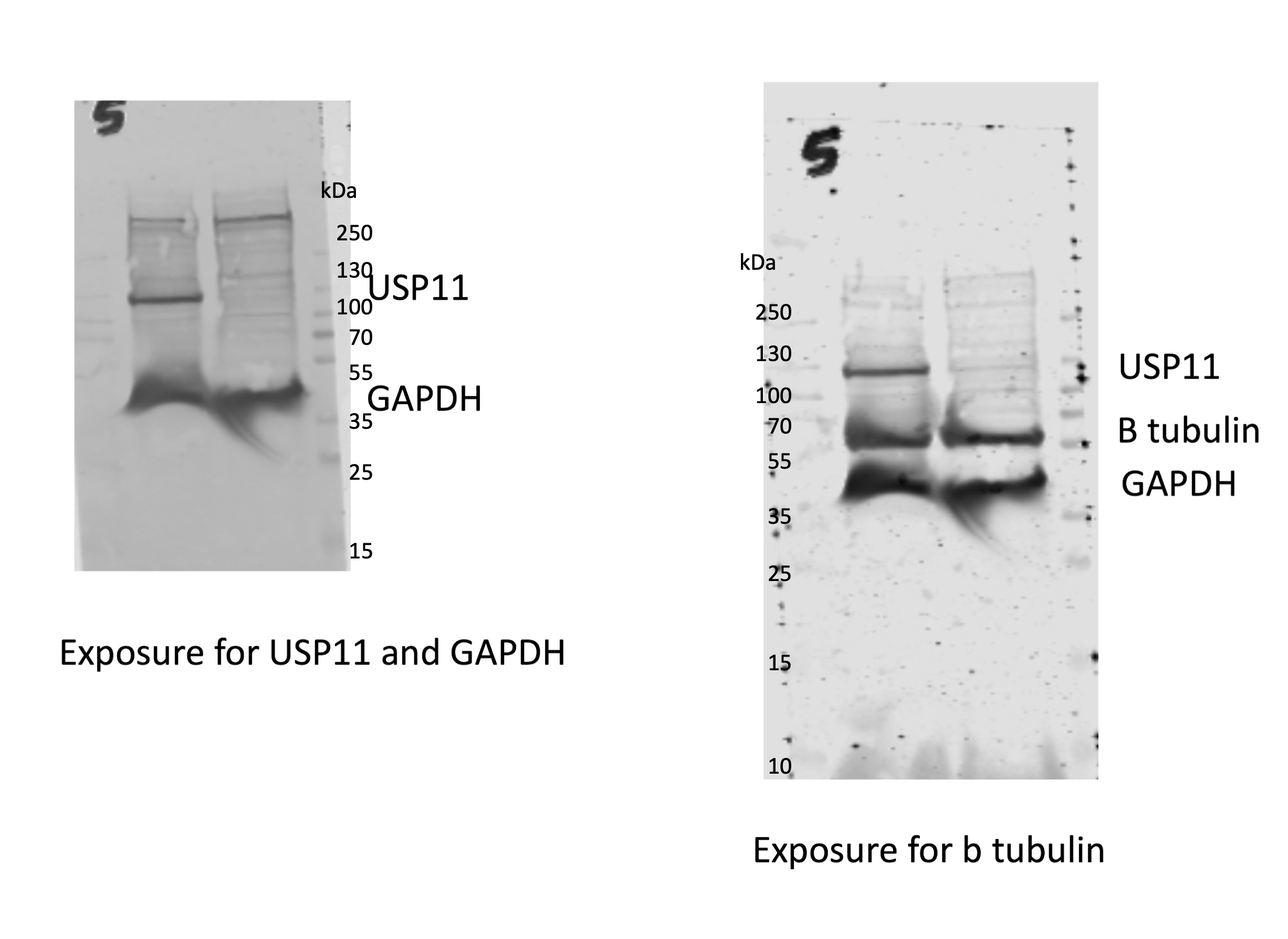

Supplement: Figure 2—figure supplement 1—source data 10. [file elife-72879-fig2-figsupp1-data10.png]

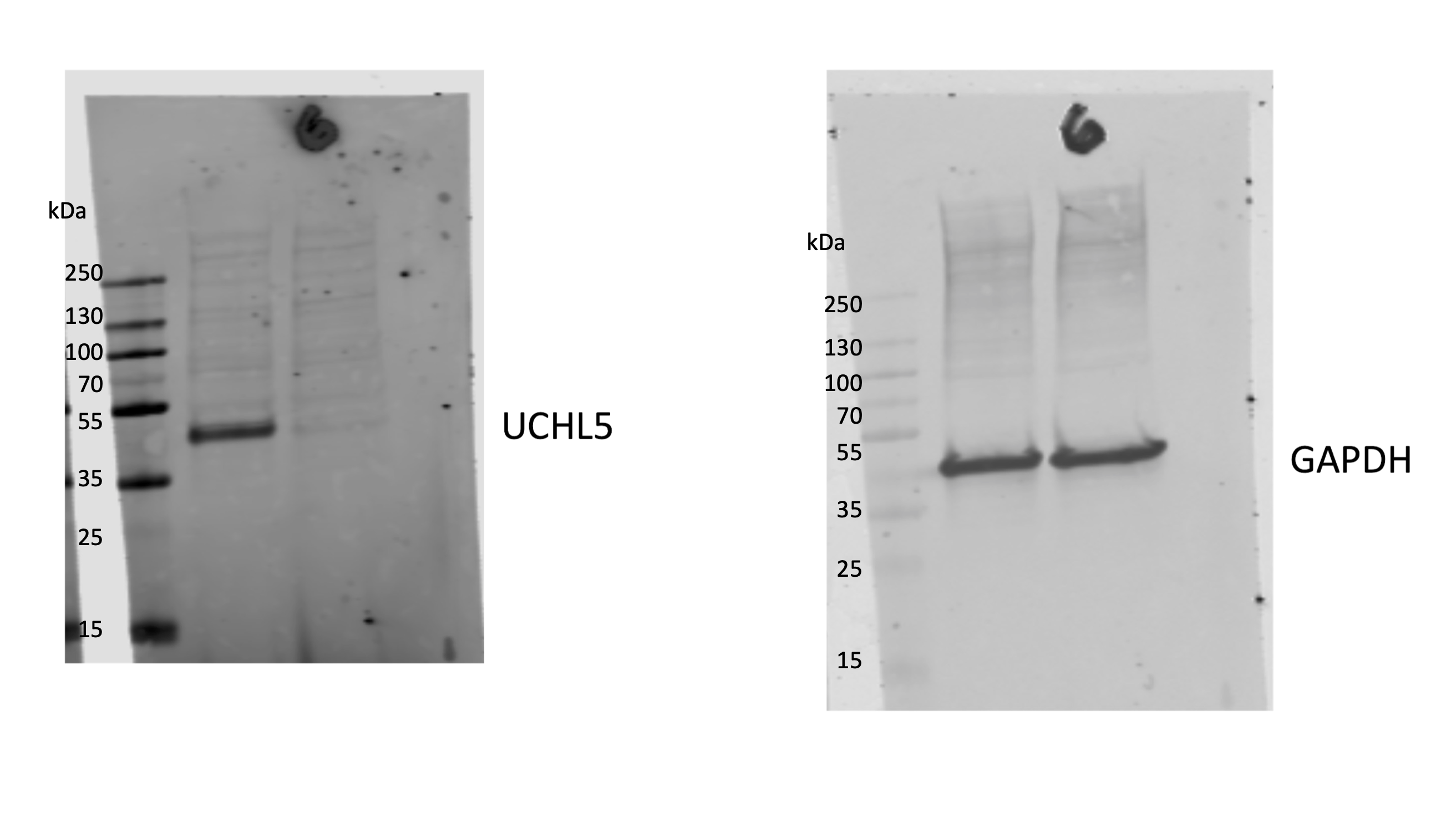

Supplement: Figure 2—figure supplement 1—source data 11. [file elife-72879-fig2-figsupp1-data11.png]
